# The *GA2ox* Gene Family in *Solanum pennellii*: Genome-Wide Identification and Expression Analysis Under Salinity Stresses

**DOI:** 10.3390/genes16020158

**Published:** 2025-01-26

**Authors:** Xianjue Ruan, Min Zhang, Tingting Ling, Xiaoyan Hei, Jie Zhang

**Affiliations:** 1 College of Landscape and Horticulture, Yunnan Agricultural University, Kunming 650201, China; xmyyyds75@163.com (X.R.); 13088058708@163.com (T.L.); hxy1838195977@163.com (X.H.); 2 College of Horticulture and Landscape Architecture, Southwest University, Chongqing 400715, China; 3 College of Foreign Languages, Yunnan Agricultural University, Kunming 650201, China; min_zhang2024@163.com; 4 College of Education and Art, Deyang Vocational College of Agricultural Science and Technology, Deyang 618500, China; 5 Key Laboratory of Vegetable Biology of Yunnan Province, Yunnan Agricultural University, Kunming 650201, China

**Keywords:** *Solanum pennellii*, *GA2ox* family, phylogenetic analysis, salt stress, expression profiles

## Abstract

Background: GA 2-oxidases (GA2oxs), a class of enzymes, inhibit the biosynthesis of bioactive gibberellins (GAs) in plants. The GA2 oxidase gene is crucial for regulating the passivation process of active GA and is widely involved in hormone signaling and abiotic stress processes. Objective/Methods: To examine the potential effects of the GA2 oxidase gene on *Solanum pennellii*, one of the important stress-tolerance wild species of tomato, a systematic analysis was performed to study the structure, phylogenetic tree, genomic locus, and upstream cis-regulatory elements of *SpGA2ox* genes. The expression patterns of the *SpGA2ox* family in various tissues were analyzed on the basis of published RNA-seq data, and the changes in *SpGA2ox* expression in the leaves of seedlings were detected under salinity stress and GA treatment by real-time fluorescence quantitative PCR. Results: We identified nine *SpGA2ox* genes in *S. pennellii*. They were located on chromosomes 1, 2, 4, 7, 8, and 10. The *SpGA2ox* family was clearly divided into three groups through phylogenetic relationship analysis, namely, five in C19-GA2ox class I, one in C19-GA2ox class II, and three in C20-GA2ox class. And cis-element analysis provided the basis for understanding the function of growth, development, hormones, and abiotic stress of GA2ox genes in *S. pennellii*. The expression patterns of the *SpGA2ox* family were different in three classes, and *SpGA2ox1* exhibited higher expression levels in the stem compared to other tissues. The expression levels of all *SpGA2ox* genes increased significantly under salt stress and decreased by treatment with GA_3_. With the largest changes in relative expression levels, *SpGA2ox3* and *SpGA2ox8* might exert key effects on the regulation of GA synthesis and the response to salt stress. Conclusions: The present study may be instrumental for further investigation into the impact of *SpGA2oxs* on responses to abiotic stress and provide potential targets for the genetic improvement of *S. pennellii*.

## 1. Introduction

Gibberellins (GAs) play a vital role in plant growth and development, as well as in responses to abiotic stress [1,2,3]. To ensure optimal concentrations of endogenous GAs, numerous enzymes are involved in their biosynthesis and inactivation. The most typical reaction of GA deactivation is 2β-hydroxylation, a reaction that is catalyzed by a group of 2-oxoglutarate-dependent dioxygenases called GA 2-oxidases (GA2oxs) [1]. The *GA2ox* genes are part of the 2OG-Fe (II) oxygenase subfamily featured by the presence of a 2OG-FeII_Oxy domain. These genes, primarily engaging in the inactivation metabolism of gibberellins, are crucial in the gibberellin metabolic pathway.

The activity of endogenous GAs can be monitored by assessing fluctuations in the expression levels of *GA2ox* genes. Consequently, these *GA2ox* genes participate in numerous developmental processes within plants. During the growth and development of *Arabidopsis thaliana*, seven *GA2ox* genes exhibit differential expression patterns [3]. *GA2oxs* enhance chlorophyll accumulation by facilitating chloroplast development in pears [4]. In mango, *GA2ox* genes may regulate plant stature by adjusting the levels of gibberellins [5]. In *Poa pratensis*, overexpression of *PpGA2oxs* reduced leaf length, increased leaf width, and reduced the content of GA_4_, which leads to dwarf traits [6].

Some research has delved into the expression of *GA2ox* genes in response to exogenous hormonal stimuli and abiotic stresses, revealing that genes involved in gibberellin oxidation play a dual role in both regulating the development and growth of plants as well as reacting to a range of abiotic stresses. In rice, exposure to low temperatures leads to the deactivation of GAs through the activation of transcription of *GA2ox* genes [7]. In maize, there was a distinct negative correlation between *GA2ox* gene expression and the levels of bioactive GAs under cold and drought conditions [8]. In potato plants, the resistance to exogenous hormones and cold stress conditions is enhanced by the overexpression of *StGA2ox1* [9]. The expression of *GA2oxs* is notably upregulated in response to exogenous GA_3_ treatment in pineapples [10] and grapes [11].

In tomatoes, 12 *SlGA2ox* family genes have been identified, with distinct *SlGA2ox* genes exhibiting tissue-specific expression patterns [12]. Modulating the expression of *SlGA2ox* genes can also impact the levels of endogenous GAs, thus influencing their growth and development. Downregulating the expression of five C19-*GA2ox* genes in tomatoes can significantly increase the content of active GA_4_, decrease the content of inactive GA_34_, reduce the number of lateral branches, and induce parthenocarpy in transgenic tomatoes [13]. Overexpressing *SlGA2ox1* in tomato fruits reduces the content of active GAs, downregulates the expression of cell expansion-related genes, leads to a decrease in cell size, and restricts fruit growth, thereby reducing the weight of the tomatoes and inhibiting seed development and germination [14].

*S. pennellii*, a wild species of tomato, is valued as a significant genetic resource for the improvement of cultivated tomatoes due to its extraordinary stress tolerance and distinctive morphology [15,16]. While the role of the gibberellin oxidase gene has been widely explored in tomatoes [17], there are relatively few studies on *GA2ox* genes in *S. pennellii*. In this research, we have identified nine *SpGA2ox* genes and classified them into three distinct subgroups. We conducted an analysis of their protein motifs, gene and protein structures, expression profiles, and chromosome distribution. In addition, qRT-PCR (real-time fluorescence quantitative PCR) was used to detect the relative expression levels of all *SpGA2ox* genes under GA_3_ treatment and their response to salinity stress. Our findings offered a comprehensive overview of the *GA2ox* gene family in *S. pennellii*, which might aid in the analysis of each gene member’s function and contribute to the genetic enhancement of tomato cultivars.

## 2. Materials and Methods

### 2.1. Identification of the GA2ox Family Genes in S. pennellii

The DNA, CDS, protein, and gff files of *S. pennellii* (annotation V2.0) were retrieved from https://solgenomics.net/ (accessed on 28 June 2024). The HMM model 2OG-Fe(II)Oxy (PF03171) extracted from the Pfam database (https://www.ebi.ac.uk/interpro/, accessed on 28 June 2024) was used as a query to search for candidate protein sequences of *SpGA2ox* in *S. pennellii*. The BLASTP method (E ≤ 1 × 10^−10^) was employed for the identification of *SpGA2ox* family members. In the present study, we mainly referred to *A. thaliana* as model plants. Seven *AtGA2oxs* in *Arabidopsis* were obtained from TAIR (https://www.arabidopsis.org/, accessed on 28 June 2024) as query sequences. The BLASTP results exceeded one hundred, with most protein sequences belonging to the 2OGD family. Since the GA degradation protein (GA2oxs) family belongs to the DOXC protein family within the 2-oxoglutarate-dependent dioxygenase (2OGD) superfamily, and GA2oxs family members share sequence similarities with proteins from other subfamilies, the BLASTP results did not distinguish between different subfamilies. We further screened *SpGA2oxs* members through phylogenetic tree analysis with published *AtGA2oxs* and *SlGA2oxs* member sequences, which was consistent with the information in the gene annotation files. Sequence alignment was performed using Tbtools software (version 2.135) [18], and then the aligned sequences were used to construct a neighbor-joining phylogenetic tree with 1000 bootstrap replicates and default parameters in MEGA v7.0.

The *SpGA2ox* gene family members in *S. pennellii* were ultimately determined through phylogenetic tree, conserved domain, and motif analyses (Table S1). The analysis of gene chromosome localization was conducted using the MG2C platform (http://mg2c.iask.in/mg2c_v2.1/, accessed on 14 July 2024). These gene family members were named according to their chromosomal positions (Table S2). The molecular weight and pI of SpGA2oxs proteins were predicted using the online tool on the ExPASy website (https://web.expasy.org/protparam/, accessed on 14 July 2024). The intron-exon structure of genes was visualized using Tbtools. The presence of conserved motifs in protein sequences was studied using MEME (Table S3 and Figure S1). The 2kb genomic DNA sequence upstream of the ATG start codon for each gene was downloaded, and the potential cis-regulatory elements in the promoter region of *SpGA2ox* family genes were identified using the Plant CARE database (http://bioinformatics.psb.ugent.be/webtools/plantcare/html/, accessed on 14 July 2024) (Table S4).

### 2.2. Expression Analysis of GA2ox Gene Family in Different Tissues of S. pennellii

To evaluate the expression patterns of the *GA2ox* genes in different tissues (stem, leaf, flower, and fruit) of *S. pennellii*, a total of 25 publicly available RNA-sequencing datasets were retrieved from NCBI (Table S5). Clean reads from each sample were then aligned against the reference genome and annotation (https://solgenomics.net/organism/Solanum_pennellii/genome, accessed on 28 June 2024) with HISAT2 [19] and StringTie v2.2.1 [20]. The parameters defined by default were used. The expression level in four tissues of each gene was calculated as the average FPKM (fragments per kilobase of transcripts per million mapped reads).

### 2.3. Plant Materials and Responses to Salt Stress

Seeds of *S. pennellii* LA0716 were sown in nutrient pots (with a diameter of 18.5 cm, a height of 16 cm, and a bottom diameter of 13 cm), which were filled with a substrate mixture of grass charcoal, vermiculite, and perlite (2:1:1). Plants were cultivated in a greenhouse at a temperature of 25 °C under a photoperiod of 16 h of light and 8 h of darkness with a relative humidity of 70% for duration of one month. During the seedling stage, each hole was irrigated with 200 mL of Hoagland nutrient solution every other day. At the initial flowering stage, salt stress treatment was applied: 100 mL of 200 mmol L⁻¹ NaCl solution was poured into the substrate per plant, and 50 mL of 100 mg L⁻¹ GA_3_ was sprayed on the leaves for treatment, with an equal amount of water used as a control. Leaf samples were collected 6 h after treatment. All samples were collected, quickly frozen in liquid nitrogen, and stored at −80 °C until RNA extraction. For analysis, three biological replicates of each sample were gathered.

### 2.4. RNA Isolation and qRT-PCR Analysis

We used an RNA kit (BIOGROUNO, Chongqing, China) to extract total RNA. All samples were reverse-transcribed into cDNA with 1 µg RNA by a reverse transcription reagent (ExonScript RT Mix with dsDNase). Because of the differences between primer design and respective SpGA2ox sequences, all primers were synthesized by Tsingke Biotech Company (Beijing, China), with the primer sequences presented in Table S6. The qRT-PCR reaction system was designed to be 50 µL, including 25 µL mix (2× SP qPCR Mix, Beijing, China), 20 μL DEPC water, 1 µL calibration dye Rox, 1 µL F-terminal primer, 1 µL R-terminal primer, and 2 µL template. The RT-PCR reaction program was completed with the following conditions: 94 °C for 20 s; 40 cycles of 94 °C for 10 s and 60 °C for 20 s; 94 °C for 15 s; and 60 °C for 60 s; 94 °C for 15 s. The analyses were confirmed in triplicate. The relative expression level of each *SpGA2ox* gene was calculated based on the comparison threshold period (2^−ΔΔCt^) method. The *UBI* gene served as the internal reference gene. The experiment was repeated to obtain three biological replicates, and three technical repeats were carried out for each biological repeat. Relative quantitative analysis was conducted on the acquired data, and statistical analysis was performed using one-way analysis of variance (ANOVA) and the least significant difference (LSD) method. Variance analysis was performed using SPSS 28 software, and the calculated values are expressed as mean ± standard deviation (SD).

## 3. Results

### 3.1. Identification of SpGA2ox Genes in the S. pennellii Genome

**Figure 1:**
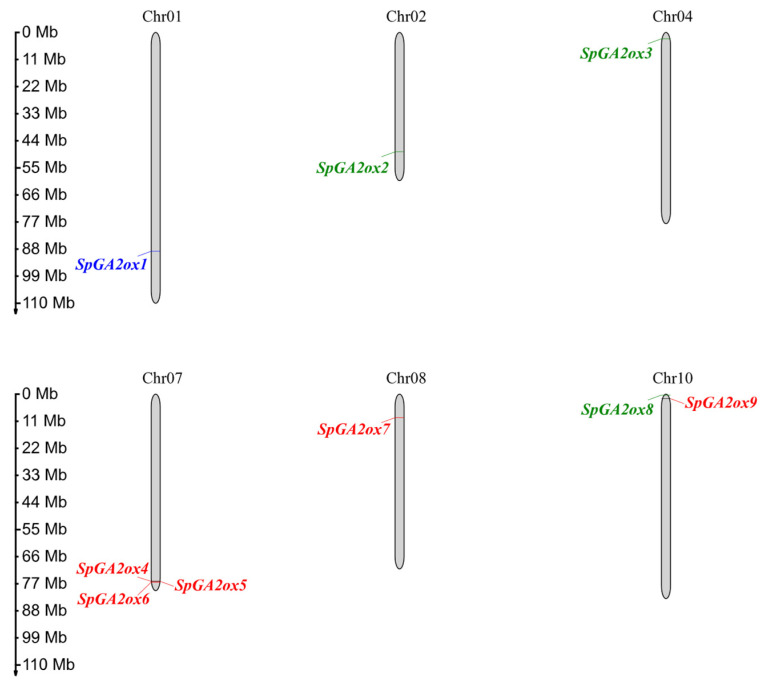
Chromosome localization of *SpGA2ox* gene family in *S. pennellii*. Red: C19-GA2ox-I. Blue: C19-GA2ox-II. Green: C20-GA2ox.

**Figure 2:**
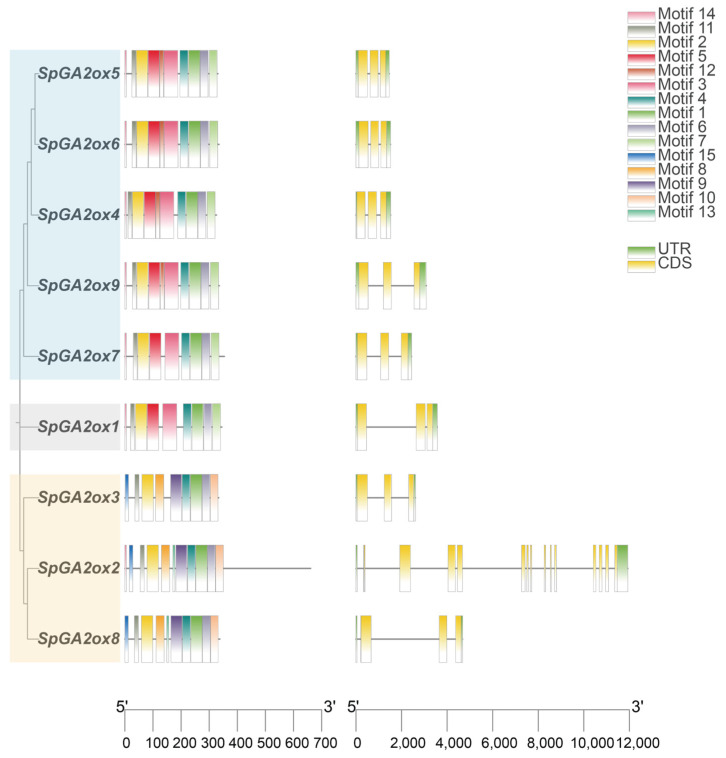
Structures of *SpGA2ox* gene family.

**Figure 3:**
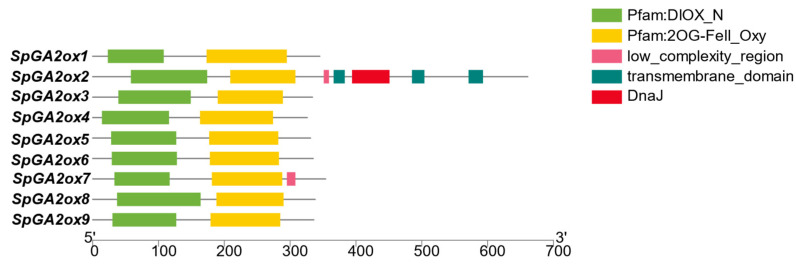
Analysis of conserved domain of SpGA2ox family proteins.

We identified nine putative *GA2ox* genes in the *S. pennellii* genome and numbered them based on their chromosomal position and their specific location on the chromosome (Figure 1). These genes were distributed on Chromosomes 1, 2, 4, 7, 8, and 10 (Figure 1). The majority of these genes were situated close to the telomeres of the chromosomes. Among them, chromosome 7 contained three *SpGA2ox* members, namely *SpGA2ox4*, *SpGA2ox5*, and *SpGA2ox6*, and the distance among them was less than 100 kb, which indicated that they formed a tandem gene cluster. Out of the nine *SpGA2ox* gene family members, all except for *SpGA2ox2* had only three exons and two introns, suggesting that the C20 genes may be more structurally complex (Figure 2). The proteins encoded by the *SpGA2ox* genes all possessed a conserved 2OG-FeII_Oxy domain and a DIOX_N domain, confirming their status as members of the 2OGD family. Additionally, the *SpGA2ox2* and *SpGA2ox7* proteins also contained low-complexity region structures (Figure 3). By analyzing the physicochemical properties of the SpGA2ox proteins, we found that these nine *SpGA2ox* genes encoded 326–661 amino acids, and the theoretical isoelectric points (pI) range from 5.33 to 9.20. In addition, the relative molecular weights (MWs) were between 36.56 and 77.77 kDa, and the instability indices (II) varied from 31.18 to 49.56, with five *SpGA2oxs* (*SpGA2ox4, 5*, *6*, *7*, *9*) having values below 40, indicating they were stable proteins, while the rest were considered unstable. Furthermore, the lipophilicity indices ranged from 75.45 to 92.99; the hydrophobicity indices were between −0.620 and −0.206; and all hydrophobicity indices were less than 0, which meant all *SpGA2oxs* were hydrophilic proteins (Table 1).

### 3.2. Phylogenetic Analysis of GA2ox Genes

**Figure 4:**
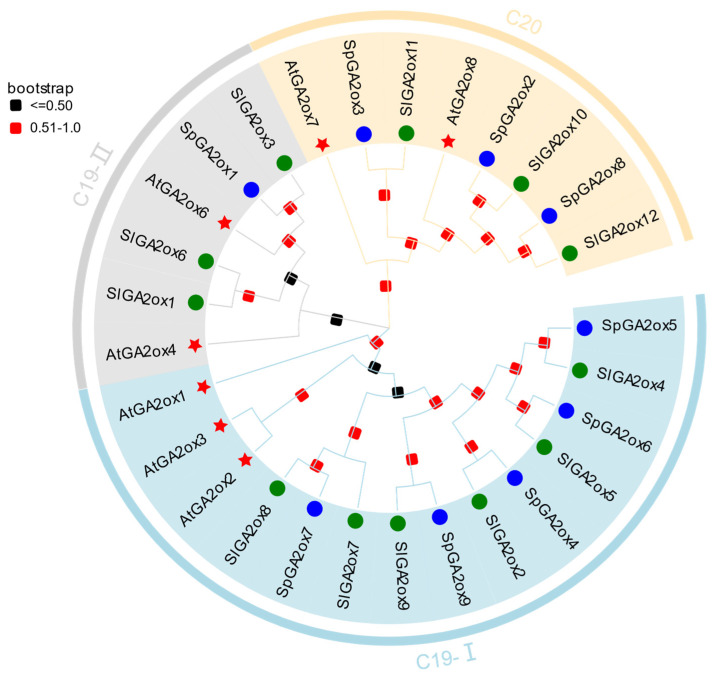
Phylogenetic tree analysis of *GA2ox* family genes in *A. thaliana* (At, red star), *S. lycopersicum* (Sl, green circle), and *S. pennellii* (Sp, blue circle).

To explore the evolutionary relationship of *GA2ox* genes, we collected 7 *Arabidopsis GA2ox* sequences and 12 tomato *GA2ox* sequences through BLASTP analysis, using the 7 *Arabidopsis GA2ox* sequences as queries. A phylogenetic tree was conducted based on their deduced protein sequences (Figure 4). The GA2ox family members were classified into three groups: C19-GA2ox-I, C19-GA2ox-II, and C20-GA2ox. The members of C19-GA2ox-I and C19-GA2ox-II were known to utilize C19-GAs as substrates, while those in C20-GA2ox are primarily active against C20-GAs. *SpGA2ox4, SpGA2ox5, SpGA2ox6, SpGA2ox7*, and *SpGA2ox9* belong to C19-I, while *SpGA2ox1* belongs to C19-II, and *SpGA2ox2*, *SpGA2ox3*, and *SpGA2ox8* belong to C20. Compared to *AtGA2oxs*, the phylogenetic relationship between *SpGA2oxs* and *SlGA2oxs* is more closely related.

### 3.3. Gene Structure and Conserved Motif Analysis of the SpGA2ox Genes

The examination of motif distribution, structural analysis, and the evolutionary relationship of *SpGA2ox* family members can be instrumental in helping us better understand the similarity and diversity within the SpGA2ox protein family members. Thus, the conserved motifs in the SpGA2ox proteins were identified through the online tool MEME. All SpGA2ox proteins contained motifs 1, 2, 4, 6, and 11, indicating that these motifs are crucial for maintaining the normal structure and function of the proteins (Figure 2). The presence of identical motifs within the same subgroup suggested that members of that subgroup might share similar biological functions. All proteins of the C19 family include motifs 1, 2, 3, 4, 5, 6, 7, 11, and 14, while all proteins of the C20 family include motifs 1, 2, 4, 6, 8, 9, 10, 11, and 15. Notably, C19-I proteins (except for SpGA2ox7) also contain motif 12, whereas C19-II proteins (SpGA2ox1) do not contain motif 12, possibly due to the closer phylogenetic relationship between SpGA2ox7 and SpGA2ox1. Interestingly, the SpGA2ox2 and SpGA2ox8 proteins of C20 also contain motif 13, and the SpGA2ox2 protein contains motif 14. These results indicated that the transcription factor binding sites of the SpGA2ox family exhibited distinct distribution characteristics within the C19 and C20 families.

### 3.4. Analysis of Cis-Acting Regulatory Elements

**Figure 5:**
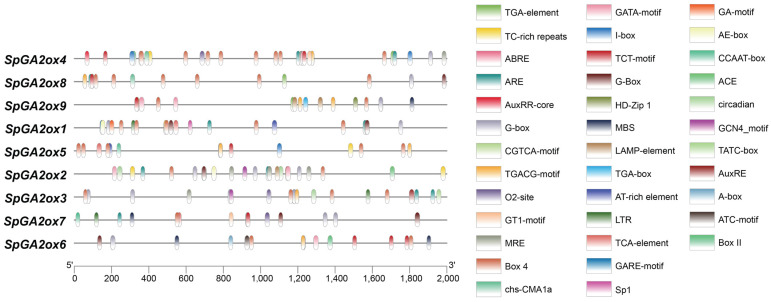
Prediction of cis-regulatory elements in the promoter of the *SpGA2ox* gene family.

Promoter regions play a crucial role in gene expression through their cis-acting regulatory elements. To investigate the response of *SpGA2ox* gene expression to hormones and various stresses, cis-acting regulatory elements were predicted and identified within the 2 kb promoter region upstream of the coding sequence of the *SpGA2ox* genes. A total of 38 cis-acting elements were screened in the promoter regions of the *SpGA2ox* family genes (Figure 5). Among them, elements related to light response were the most common in the promoters of *SpGA2ox* genes, accounting for the largest proportion (34.95%), including G-box, Box 4, ACE, GT1-motif, Sp1, MRE, ATC-motif, chs-CMA1a, GA-motif, Gap-box, GATA-motif, I-box, LAMP-element, TCT-motif, AE-box, and TCT motifs. Additionally, there were many cis-regulatory elements related to plant hormone responses, such as TATC-box, GARE-motif, TGA-element, TCA-element, CGTCA-motif, TGACG-motif, and ABRE. Notably, the promoters of *SpGA2ox1* and *SpGA2ox3* contained the GARE-motif and TATC-box, which were cis-regulatory elements responsive to GAs, respectively. Furthermore, cis-regulatory elements related to external or environmental stress responses were also present, including LTR (low-temperature response element), MBS (MYB binding site associated with drought resistance), ARE (required cis-regulatory element for anaerobic induction), and defense response elements TC-rich repeats (cis-regulatory elements involved in defense and stress responses).

These results suggested that the expression of *SpGA2ox* family genes may be regulated by cis-elements associated with light responsiveness, plant hormones, defense signal transduction, and various stresses during the growth and development of *S. pennellii*, indicating that the *SpGA2ox* family genes might play an important role in responding to GAs and salt stress.

### 3.5. Transcriptional Profile of SpGA2ox Genes in Different Tissues

**Figure 6:**
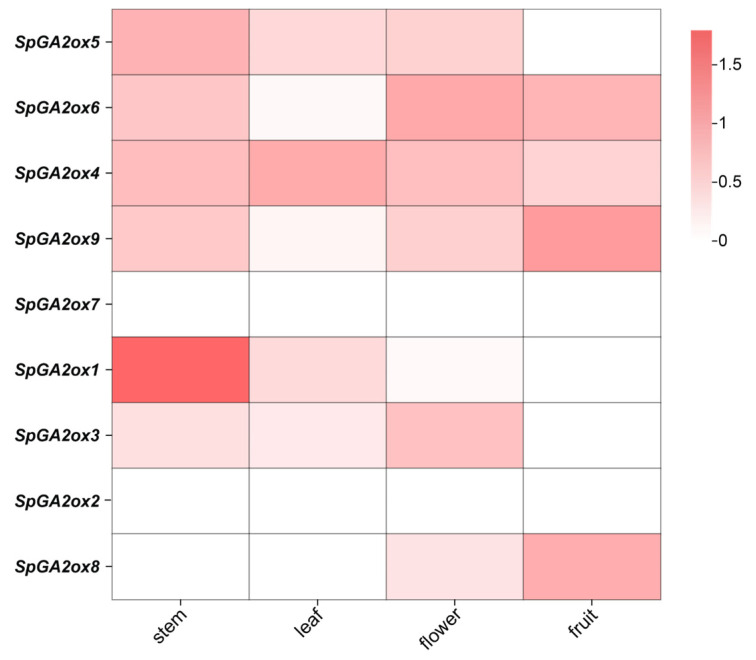
Expression heatmap of *SpGA2ox* genes in different tissues (The color scale represents log 2 (FPKM + 1) values).

To examine the expression profile of *SpGA2ox* genes in different tissues, we analyzed their transcript levels in four tissues (flower, fruit, leaf, and stem) (Figure 6). The C19-I class genes, including *SpGA2ox4, SpGA2ox5, SpGA2ox6*, and *SpGA2ox9*, with the exception of *SpGA2ox7*, were expressed in all four tissues. *SpGA2ox1* exhibited higher expression levels in the stem compared to other tissues. The three C20 class genes, *SpGA2ox2, SpGA2ox3*, and *SpGA2ox8*, showed low and uneven expression across the four tissues.

### 3.6. Expression Response of SpGA2ox Genes to NaCl and GA_3_ Treatment

The *SpGA2ox* family genes are involved in encoding GA2-oxidase genes in *S. pennellii*, which play a crucial role in the metabolism of active gibberellins (GAs). They are also widely involved in the signal transduction of hormones such as GA, abscisic acid (ABA), and jasmonic acid (JA), as well as in responses to abiotic stresses. To investigate the response of key GA degradation genes in *S. pennellii* leaves to salt stress and the alleviation of salt stress by gibberellins, we measured the expression levels of the *SpGA2ox* family genes across various treatment groups.

**Figure 7:**
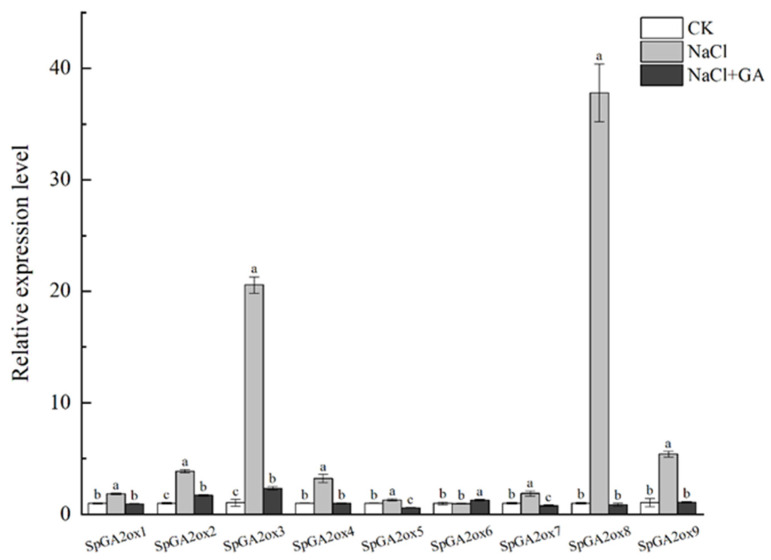
Expression patterns of *SpGA2ox* genes in leaf tissues under salt stress and GA_3_. Each treatment is presented with the mean ± SD (*n* = 3). Different lowercase letters denote significant differences (*p* < 0.05).

In this experiment, *S. pennellii* seedlings were treated with 200 mM NaCl and a combination of 100 mg L^−1^ GA3 and 200 mM NaCl for 6 h. Then, samples were taken, and the expression levels relative to a water control were determined using RT-qPCR (Figure 7). The results showed that under salt stress, the expression levels of the *GA2ox* genes in *S. pennellii* leaves increased significantly, except for *SpGA2ox6*. Moreover, when 100 mg L^−1^ GA_3_ was applied simultaneously with salt stress, the expression levels of the *SpGA2ox* genes significantly decreased compared to the salt stress group alone. Notably, *SpGA2ox3* and *SpGA2ox8* exhibited larger changes in relative expression under the combination of salt treatment and 100 mg L^−1^ GA_3_. These results suggested that the *SpGA2ox* genes might play an important role in regulating the salt stress response in *S. pennellii* and might be involved in the regulatory process by which gibberellins alleviate salt stress in tomatoes.

In summary, the expression levels of the *SpGA2ox* family genes increased significantly under salt stress, except for *SpGA2ox6*, with *SpGA2ox3* and *SpGA2ox8* showing the largest changes in relative expression. However, when gibberellins were applied concurrently with salt treatment, the expression levels of the *SpGA2ox* family genes decreased compared with the salt stress group, leading to a significant reduction in the amount of GA2-oxidase encoded by *SpGA2ox*. This reduction increased the formation of complexes among active gibberellins, gibberellin receptors, and DELLA proteins, inhibited the accumulation of DELLA proteins, promoted gibberellin signal transduction, and ultimately promoted plant growth and development. Therefore, the experimental results provided a theoretical basis for the alleviation of salt stress in *S. pennellii* by gibberellins.

## 4. Discussion

Plants synthesize active endogenous gibberellins (GAs) through the enzymes *GA20ox* and *GA3ox* and inactivate them through GA2ox enzymes to regulate the dynamic balance of GA levels within the plant, thereby controlling normal growth and development [21]. In the present study, we identified nine *SpGA2ox* genes in the *S. pennellii* genome (Figure 1), and they showed similarities to findings in *A. thaliana* and tomato genomes [3,12]. According to the phylogenetic analysis of the genes, *SpGA2ox1*, *SpGA2ox4*, *SpGA2ox5*, *SpGA2ox6*, *SpGA2ox7*, and *SpGA2ox9* belong to the C19 family, while *SpGA2ox2*, *SpGA2ox3*, and *SpGA2ox8* belong to the C20 family, which is similar to the findings of the tomato *SlGA2ox* gene family in the 2OGD family [12]. However, unlike previous studies, this experiment further classified the C19 family. Through motif and phylogenetic analysis, it was found that there were significant differences in the conserved motif sequences among different subfamilies of *SpGA2ox*. Specifically, C19-I class proteins (except for *SpGA2ox7*) also contained motif 12, while C19-II class proteins (*SpGA2ox1*) did not contain motif 12.

The analysis of cis-regulatory elements indicated that the *SpGA2ox* family genes were widely involved in light signal transduction, plant hormone signal transduction, and stress response processes, with functional differences among family members, which was consistent with findings in crops such as potato [22] and pineapple [10]. In this experiment, the promoters of the *SpGA2ox* family genes were also analyzed, and the results showed that these promoters contained cis-regulatory elements involved in stress response, such as TC-rich repeats; elements involved in the response to the stress hormone abscisic acid (ABA), like ABRE; and elements involved in the response to jasmonic acid (JA), such as TGACG-motif. Additionally, the promoters of *SpGA2ox1* and *SpGA2ox3* contained cis-regulatory elements responsive to gibberellins, GARE-motif, and TATC-box, respectively. This suggested that the family genes were related to stress response and plant hormone signal transduction processes.

Gene expression patterns offer a preliminary indication of gene function, and tissue-specific expression patterns are crucial for understanding the roles of *GA2ox* genes in modulating plant architecture [1,23]. Transcriptome analyses indicated that only a minor fraction of the *SpGA2ox* genes exhibited high expression levels in plants. This is because GA2ox, acting as an enzyme that degrades gibberellin activity, only upregulates its expression when there is an excess of gibberellins in plants (Figure 6).

The qPCR results showed that under salt stress, the expression levels of the *SpGA2ox* family genes in *S. pennellii* leaves increased significantly, except for *SpGA2ox6* (Figure 7). Moreover, when 100 mg L^−1^ GA_3_ was applied simultaneously with salt stress, the expression levels of the *SpGA2ox* family genes significantly decreased compared to the salt stress group, with *SpGA2ox3* and *SpGA2ox8* showing the largest changes in relative expression. These results implied that the family genes played an important role in regulating the salt stress response in *S. pennellii* and that the *SpGA2ox* family might be involved in the regulatory process by which gibberellins alleviate salt stress in tomatoes. The expression analysis of abiotic stress in this experiment was largely consistent with that of the *AhTPS* family genes under cold stress in cultivated peanuts [24]. However, there was a difference in that the previous study measured expression levels at various time points under cold stress, whereas this experiment measured expression levels after 6 h of salt stress. Furthermore, the expression analysis under salt stress in this experiment also aligned with that of the *StGA2ox* family genes under low-temperature stress in potatoes, but the expression analysis of response to gibberellins was different [22]. The possible reason was that in the previous research, the family genes’ response to gibberellins was examined with only GA_3_ applied, whereas in the current study, we investigated the role of gibberellins in salt stress from the perspective of the *GA2ox* family genes under the combination of salt treatment and GA_3_.

## 5. Conclusions

In the present study, a total of nine *SpGA2ox* genes were identified in the *S. pennellii* genome, and these *SpGA2ox* genes were categorized into three groups. The expression patterns of *SpGA2ox* genes varied across different tissues, demonstrating strong spatiotemporal specificity. The expression analysis results of *SpGA2ox* genes in response to salt stress showed that some *SpGA2ox* genes were involved in the regulation of stress tolerance. Our research will lay a foundation for the identification of *SpGA2ox* gene family members and the functional mechanism by which *SpGA2ox* genes are involved in abiotic stress response.

## Figures and Tables

**Table 1:** Characteristics of SpGA2ox family genes and their encoded proteins.

Sequence ID	Gene ID	Size (AA)	MW (Da)	pI	Instability Index	Aliphatic Index	Grand Average of Hydropathicity
*SpGA2ox1*	*Sopen01g030980.1*	345	38,745.61	6.71	43.87	75.45	−0.392
*SpGA2ox2*	*Sopen02g024840.1*	661	77,767.94	9.2	49.56	75.67	−0.62
*SpGA2ox3*	*Sopen04g003840.1*	334	38,431.04	6.76	41.33	83.17	−0.331
*SpGA2ox4*	*Sopen07g030010.1*	326	36,563.69	5.93	39.35	86.1	−0.247
*SpGA2ox5*	*Sopen07g030070.1*	331	37,233.79	8.53	32.34	88.58	−0.206
*SpGA2ox6*	*Sopen07g030080.1*	335	37,660.98	6.13	39.5	83.16	−0.264
*SpGA2ox7*	*Sopen08g007060.1*	354	39,681.71	8.35	38.79	92.99	−0.244
*SpGA2ox8*	*Sopen10g001350.1*	338	39,183.48	5.33	48.02	77.84	−0.375
*SpGA2ox9*	*Sopen10g003490.1*	336	38,141.99	7.6	31.18	89.02	−0.255

## Data Availability

The original contributions presented in the study are included in the article/Supplementary Materials, further inquiries can be directed to the corresponding author.

## Supplementary Materials

The following supporting information can be downloaded at: https://www.mdpi.com/article/10.3390/genes16020158/s1, Figure S1: Location of motifs for *SpGA2ox* gene family; Table S1: *SpGA2ox* family members after being blastp filtered; Table S2: Chromosome localization of SpGA2ox gene family in *S. pennellii*; Table S3: Information of motifs for *SpGA2ox* gene family; Table S4: Prediction of cis-regulatory elements in the promoter of the SpGA2ox gene family; Table S5: The summary of RNA-seq datasets; Table S6: The primers used for qRT–PCR in the study.
