# Supplementary material for: The GA2ox Gene Family in Solanum pennellii: Genome-Wide Identification and Expression Analysis Under Salinity Stresses
Source: Genes (Basel). 2025 Jan 26;16(2):158. doi: 10.3390/genes16020158 (PMC11855036; doi:10.3390/genes16020158)
Supplement: Supplementary file 1 [file genes-16-00158-s001.zip › Figure S1 Location of motifs for SpGA2ox gene family.pdf]

Name	*p*-value
SpGA2ox1	2.75e-178
SpGA2ox2	5.02e-220
SpGA2ox3	1.88e-184
SpGA2ox4	1.05e-282
SpGA2ox5	1.32e-293
SpGA2ox6	6.20e-284
SpGA2ox7	1.67e-202
SpGA2ox8	1.78e-220
SpGA2ox9	4.66e-268

Motif Locations

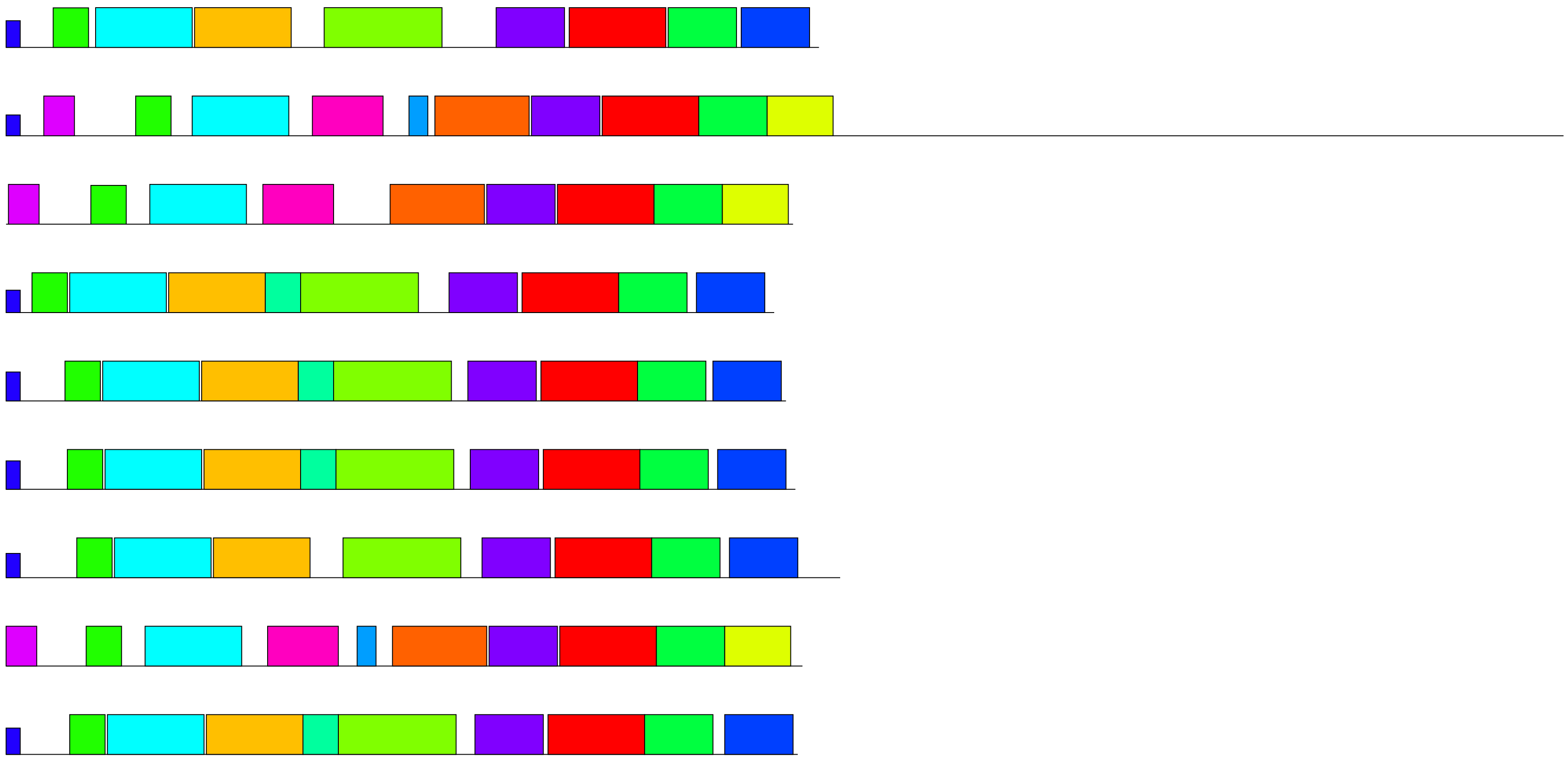

Motif	Symbol	Motif Consensus
1.		KDGKWISVPPDQNSFFVNVGDSLQVMTNGRFKSVKHRVLTN
2.		IVKACEEFGFFKVVNHGVPMEFJSKLESEAIKFFSSPLSEK
3.		DYVSAVKKMSCEILEMLAEGLKIHPTNVFSKLLMDEKSDSVFRLNHYPPC
4.		SKNLIGFGEHTDPQIJSILRSNNTSGLQI
5.		KAGPADPFGYGNKKIGQNGDIGWVEYILLSTNSEFNYQKFA
6.		SVKSRLSMIYFGGPPLDEKIAPLPSLMEY
7.		LYKEFTWFEYKKSAYKTRLADNRLVLFEK
8.		GSYRWGTPTATCLRQLSWSEAFHVPLTDIS
9.		LARDLAEILGEKLNYKDDYFKETCLPNTCYLRMNRYPACP
10.		KKFTFREFRQQVQEDVKSFGYKVGLPRF
11.		NEIPLIDLSKPNSKN
12.		SILGVNPENIRAAVN
13.		TMEZFATT
14.		MVVLTK
15.		MDESPDPPFVETY
