# Supplementary material for: The GA2ox Gene Family in Solanum pennellii: Genome-Wide Identification and Expression Analysis Under Salinity Stresses
Source: Genes (Basel). 2025 Jan 26;16(2):158. doi: 10.3390/genes16020158 (PMC11855036; doi:10.3390/genes16020158)
Supplement: Supplementary file 1 [file genes-16-00158-s001.zip › Table S6. The primers used for qRT–PCR in the study.docx]

Table S6 The primers used for qRT–PCR in the study.

| Genes | Primers |
| --- | --- |
| *SpGA2ox1-q-F* | 5' GGGACTCCTTTCAGGCGTT 3' |
| *SpGA2ox1-q-R* | 5' CCAAGCGTCAAGAGCAGGA 3' |
| *SpGA2ox2-q-F* | 5' CTTCTCACAGCAGCCAAACCA 3' |
| *SpGA2ox2-q-R* | 5' ATCCTTTTCCGATTCTTATGTCGTC 3' |
| *SpGA2ox3-q-F* | 5' GGTGTGAGCCTTGAGTTGCT 3' |
| *SpGA2ox3-q-R* | 5' TCCACCATGACATCCCTTAAAGT 3' |
| *SpGA2ox4-q-F* | 5' TTGGGTCGAACACATCCTCGT 3' |
| *SpGA2ox4-q-R* | 5' ACCGAATGTTTTCTGGATTGACACC 3' |
| *SpGA2ox5-q-F* | 5' TCCAACAACACTTCCGGTCT 3' |
| *SpGA2ox5-q-R* | 5' GGTGCTATCTTTTCACTCAATGGT 3' |
| *SpGA2ox6-q-F* | 5' AAGCACAGGGTACTGACAAACA 3' |
| *SpGA2ox6-q-R* | 5' GGTGCTATCTTTTCACTCAATGGT 3' |
| *SpGA2ox7-q-F* | 5' AATCACGGTGTTCCTGATGAAACGA 3' |
| *SpGA2ox7-q-R* | 5' ACCGGCTTTGTCCTTGTCACA 3' |
| *SpGA2ox8-q-F* | 5' GCAGCCTCAGCTCTACCAT 3' |
| *SpGA2ox8-q-R* | 5' AAGTCACTATCAGTATGTGGCATT 3' |
| *SpGA2ox9-q-F* | 5' TGGTGATTCATTGCAGGTGATG 3' |
| *SpGA2ox9-q-R* | 5' AGTGATGCCAATGGTGCTATCT 3' |
| *UBI-q-F* | 5' TCGTAAGGAGTGCCCTAATGCTGA 3' |
| *UBI-q-R* | 5' CAATCGCCTCCAGCCTTGTTGTAA 3' |
